# Possible Mechanisms of Relations between the Thermal Neutrons Field and Biosphere

**DOI:** 10.1155/2020/2175296

**Published:** 2020-08-11

**Authors:** Anton V. Syroeshkin, Irina V. Tarabrina, Mariya A. Morozova, Alla V. Marukhlenko, Igor A. Zlatskiy, Mariya P. Makarova

**Affiliations:** ^1^Department of Pharmaceutical and Toxicological Chemistry, Peoples Friendship University of Russia (RUDN University), Miklukho-Maklay Str.6, Moscow 117198, Russia; ^2^Laboratory of Applied Biotechnologies, State Institute of Genetic and Regenerative Medicine NAMS of Ukraine, 67 Vyshgorodska Str., Kyiv 04114, Ukraine

## Abstract

This paper proposes some results concerning the interaction of living matter of different organization levels (prokaryotes and eukaryotes) with the flux of thermal neutrons. The phenomenon of the virtual neutron trap was tested during the passage of thermalized neutrons from the Pu-Be couple through a flat layer of *E. coli* suspension. We have studied the metabolic characteristics of *A. salina* cysts before and after artificial neutron flux exposure. It has been demonstrated that the concentrations of some metals in samples of alive and dead cysts irradiated with an artificial flow of thermal neutrons are not equal. The content of Mn in alive *A. salina* samples has increased more than ten-fold after their interaction with neutron flux, while the amount of As decreased by a factor of two after exposure. Levels of other elements (Al, Cr, Ni, Cu, Cd, and Pb) did not show any significant difference. Trace element composition of cysts was assessed using the method of atomic absorption spectroscopy with electrothermal atomization and the Zeeman background correction.

## 1. Introduction

Neutrons do not carry any electric charge, what allows them to freely penetrate deep into the atoms and reach the nuclei. In elastic scattering on nuclei of C, N, O, and other elements that make up the tissues, the neutron loses 10–15% of the energy. When colliding with almost equal hydrogen nuclei (protons), the energy decreases, on average, twice, being transferred to a recoil proton. Strongly ionizing protons are formed because of elastic neutron scattering. Upon neutron absorption, atomic nuclei become unstable and, then, decay generating direct and indirect ionizing radiation—protons, *ά*-particles, and *γ*-radiation photons [1]. With such nuclear reactions, radioactive isotopes of elements can be formed. The recoil nuclei that arise during nuclear transformations also ionize the substance. Thus, with neutron irradiation, the final biological effect is associated with ionization produced indirectly by secondary particles or photons.

The influence of the neutron flux on living organisms depends on the neutron energy and its intensity. Fast neutrons and a high-intensity neutron flux cause, for the most part, destructive changes and mutations in living organisms. The question of the effect of thermal neutrons in sufficiently high doses on living organisms is quite thoroughly consecrated in the literature [2]. Streams of thermal neutrons of low intensity also possess bio efficiency. The works [3, 4] note the correlation between the flux of natural origin thermal neutrons and the modulations of functional indicators of blood underlying the nonspecific immunoreactivity of the organism. The flux of thermal neutrons causes the dispersion of cellular associates of *E. coli* cells (strain DH5*α*), what indicates the stimulating (in physiological terms) effect [5]. Irradiation of *Artemia salina* cysts with a flow of thermal neutrons of different intensities (from 7 to 10^5^ n/(s·m^2^)) causes change in the spin-spin relaxation time of protons (NMR-spin echo method), which indicates a change in the functional state of the cell [6].

The goal of this study is to show that living organisms as a heterogeneous medium can act with thermalized neutrons becoming a kind of neutron trap. Assuming that the increase in thermal neutron capture is due to the structural features of the interfacial layers, the effect of a weak thermal neutron flux on the metabolism of metals in living cells should be expected. So, this work also proposes some results on the investigation of the background thermal neutron flux effect on metabolic rates of living systems on the example of the microelement composition of *A. salina* diapausing cysts.

## 2. Materials and Methods

The formation of a virtual neutron trap that allows one to accumulate thermal neutrons and release them with a given time delay can be carried out according to the following scheme: there should be a heterogeneous absorber in the path of the neutron flux with an inhomogeneous electric field in the bulk phase and external and internal interphase zones with a voltage above 10^5^ V/cm (artificial or natural sources of electric field). The stationary (averaged) transmembrane potential on the biomembrane can reach 10^7^ V/cm and, at local values, up to 10^10^ V/cm [7, 8]. For this, a bacterial suspension and a powdery preparation of *A. salina* cysts were chosen.

The experiment is set up on irradiating cysts of *A. salina* by neutron flux with consequent elemental analysis.

Brine shrimp *Artemia salina* can be considered a model animal extremophile offering a unique suite of adaptations. Under extremely critical environmental conditions, *Artemia* takes refuge by producing a highly resistant encysted gastrula embryo (cyst) capable of severe dehydration enabling an escape from population extinction. Cysts can be viewed as gene banks that store a genetic memory of historical population conditions [9]. These cysts are metabolically inactive and do not further develop as long as they are kept dry. Cysts are arguably the most resistant of all animal life history forms to environmental stress [10].

In this study, we used dry activated *Artemia* cysts from Great Yarovoye Lake, Altai Krai, Russia (ARSAL, Russian Federation, 2005). The amount of amino acids was 47% and above and that of lipids was 15% and above, and the humidity level was not more than 8%. Cysts were killed by heating in a desiccator in a glass container at a constant temperature (+150°С) for 9 hours; cysts were considered dead when they had no germination while there were no external changes.

The irradiation unit consisted of a neutron source INK1-06 (^252^Cf, activity—1.42·10^4^ n/c ± 8%); neutron moderator (polyethylene); and a cuvette for *A. salina* cysts (plexiglass, height—18 cm, diameter—4.8 cm, and wall thickness—2 mm), coaxially encompassing the ^3^Не-neutron counter of type SI14N. The total flux density of neutrons after thermalization was about 20 n/(c·cm^2^)—1000 times greater than the density of the background flux above the earth's surface. The equivalent dose rate at the location of the samples was about 1.3 *μ*Sv/hour, which exceeded the natural radiation background by not more than an order of magnitude. So, from the point of view of ionizing effects on living cells, the analysed neutron irradiation can be considered weak. Samples of alive and dead cysts were irradiated for 1 hour.

All samples of *A. salina* cysts were subjected to acid mineralization. The metal content was determined with an atomic absorption spectrometer “SpectrAA-800” (Varian, USA) with electrothermal atomization and the Zeeman background correction.

For each type of samples, 3 parallel experiments were carried out, including the stages of sample preparation and measurement. In fact, inside each cycle, 15 measurements were taken for repeatability using the AAS method. To assess the significance of difference between two groups, alive cysts and irradiated alive cysts, the Mann–Whitney *U* test (Wilcoxon rank sum test) was used, with software OriginPro (OriginLab, USA).

### 2.1. Setting Up the Experiment on Irradiating *E. coli* Cell Culture by Neutron Flux

**Figure 1:**
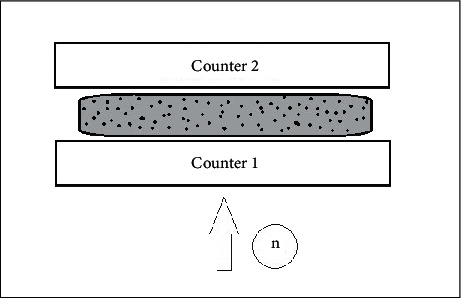
Scheme of the installation for the study of the neutron flux impact on the cell culture.

The general scheme of the installation is shown in Figure 1. The neutron flux from the source (plutonium-beryllium couple) alternately passed through the *E. coli* cell culture (strain DH5*α*). The culture flask was placed between two neutron counters (^3^He-neutron counter type C14N). The first counter recorded the neutron flux coming directly from the radiation source, and the data of the second counter allowed estimating the time delay of the neutron flux in the cell culture. During the study, the environmental conditions were changed using water (Milli-Q) as a solvent, and in one of the variants of the experiment, 2,4-dinitrophenol (2,4-DNP) was used, which made it possible to estimate the neutron delay time in a heterogeneous system in the absence of the membrane potential effect of living cells. The total number of repetitions was 9.

Growing a bacterial culture (suspension): one colony of *E. coli* cells (strain DH5*α*) was seeded in 10 ml of the LB medium (pH 7.5) containing 10% peptone, 0.5% yeast extract, and 1% NaCl with an ampicillin concentration of 100 mg/ml. Reseeding of cells was carried out using a pasteurized flame platinum loop, and the culture was grown overnight at 37°C with aeration. Cells were grown to an optical density of 0.6 (*λ* = 600 nm). The resulting volume of the bacterial suspension was placed in a Falcon type tube (500 ml).

## 3. Results and Discussion

The phenomenon of the virtual neutron trap was tested during the passage of thermalized neutrons from the Pu-Be couple through a flat layer of *E. coli* suspension.

**Figure 2:**
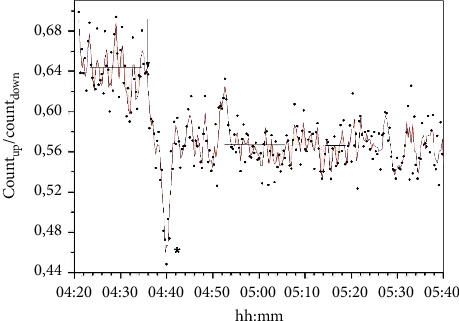
The kinetics of the formation of a virtual neutron trap when a suspension of *E. coli* is introduced into the neutron flux (the moment of introduction is indicated by the arrow). The concentration of cells in the suspension was 10^9^ cm^−3^. Neutron flux was measured using two ^3^He counters installed on the path of the channeled thermalized neutron flux before (counter down) and after (counter up) falcons with the suspension along the normal to the flux. (DNP) = 1 mM.

The introduction of falcons in the neutron flux areas led to a decrease in the thermal neutron flux density, as shown in Figure 2. The peculiarity of this decrease was the presence of a kinetic failure lasting 1–3 minutes. The indicated kinetic failure degenerated when dinitrophenol (DNP), the uncoupler of oxidative phosphorylation process, was introduced into the medium, which leads, as is known [11], to the discharge of transmembrane electrochemical potential (see Table 1).

As for *A. salina*, we observed a certain difference in the count of neutrons, passed through the installation with samples of alive and dead cysts: living cysts showed 4% higher neutron flow than the dead. Neutron irradiation of *A. salina* alive cysts also activated their germination rate when seeding in seawater: by the 42^nd^ hour of incubation, the number of nauplii formed from irradiated cysts exceeded that grown from the nonirradiated by a factor of 4 [12].

Assuming that the increase in thermal neutron capture is due to the structural features of the interfacial layers, the effect of a weak thermal neutron flux on the metabolism of metals in living cells should be expected.

**Figure 3:**
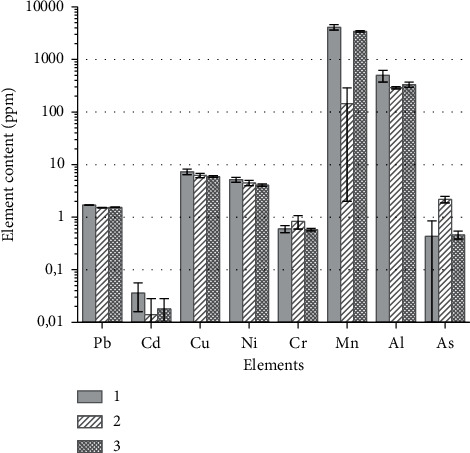
Changes in the elemental profile of *A. salina* cysts with neutron irradiation and thermal death (1—test of living cysts; 2—test of living cysts, which were exposed with neutrons for an hour; 3—test of dead cysts). The content of elements in the test of dead cysts is averaged for unirradiated and irradiated samples. All data on the content of elements have the dimension of *μ*g element per g of cyst mass; the error bars represent the standard deviation of measurements for 15 runs in 3 parallel experiments (*n* = 45).

So, in addition to the biological effect of accelerated development of *A. salina* cysts, hourly neutron irradiation led to some metabolic effects. The content of Mn in alive *A. salina* samples has increased more than ten-fold after their interaction with neutron flux (see Figure 3), while the amount of As was significantly two-fold decreased after exposure. The levels of other elements (Al, Cr, Ni, Cu, Cd, and Pb) in alive cysts did not show any significant difference.

According to our data, the specific change in element profiles of *A. salina* cysts followed by a biological response also appears after other physical action. Particularly, with a monthly cold (−20°С) activation of cyst germination, the copper-zinc ratio significantly changes equimolar, and at the same time, values for the rest of the elements stay unchanged [6–13].

The mechanisms of the influence of thermal neutrons on organisms can consist in promotion of oxidoreductase processes due to the generation of electrons during the decay of a free neutron (*n* ⟶ *p* + *e*, half-life is 11 minutes) and when a thermal neutron is captured by protium (*p* + *n* ⟶ D). The cross section of radiative capture of thermal energy neutrons by protium is up to 1/4 of the total scattering cross section. As a result, there are phonon oscillations. It has been experimentally found that phonon oscillations can be caused by irradiation of even ultrasmall thermal neutron fluxes [14]. It can be assumed that, in a living system, there is a similar mechanism that leads to the generation of membrane potential, and a biological response is observed. Perhaps, in this case, thermal neutrons act as a stimulus in relation to the internal environment of the resting biosystem, thereby helping it stay in suspended animation for a long period. At the same time, the system does not need to consume its own energy.

## 4. Conclusions

The registered phenomenon of a “neutron trap” in the *E. coli* culture medium, which disappeared after DNP was added, and observed differences in the interaction of alive and dead *A. salina* cysts with neutrons, in our opinion, are due to the presence of transmembrane potentials (Δ*μ*). As Δ*μ* can reach values up to 250 mV, an electric field gradient (Е) of at least 500 kV/cm is formed on biomembranes with a thickness of about 5 nm. Considering surface potentials, the values of E on some parts of the membrane can be by 2–3 orders of magnitude higher. This leads to both chemical effects (such values of E meet the conditions for the realization of the second Wien's effect, ultrahigh ions mobility due to the “loss” of the hydration shell) and to the polarization of nuclei that, in turn, change the nature of the interaction between matter and neutrons [15]. It should be emphasized that, in this study, we used the flux density of thermal neutrons close to the natural background values. The presence of biological and physiological (see Figure 3) responses to weak neutron fluxes allows to assume that neutrons are one of the main “mediators” which provide living organisms feeling a whole range of astrogeophysical events [5].

## Figures and Tables

**Table 1:** Thermal neutron flux density (s^−1^·cm^−2^) in the upper counter in the presence or absence of transmembrane electrochemical potential in cells of the *E. coli* suspension. The minimum values were measured 3–9 times in the area indicated by an asterisk in Figure 2. The median flux values were measured for the stationary site after making the cell culture (indicated by the line in Figure 2).

Conditions	Minimum values, s^−1^·cm^−2^ (^*∗*^in Figure 2)		Median values, s^−1^·cm^−2^	
	Variability	Variability
+Δ*μ*_H_+	3400	90	3830	186
−Δ*μ*_H_+ (+1 mM DNP)	3730	90	3790	178

## Data Availability

The data used to support the findings of this study are available from the corresponding author upon request.
